# Carbonyl-to-nickel exchange on a lactam skeleton

**DOI:** 10.1038/s42004-023-01022-0

**Published:** 2023-10-12

**Authors:** Huijuan Guo

**Affiliations:** Communications Chemistry, https://www.nature.com/commschem

## Abstract

Molecular skeletal editing has a wide range of applications in late-stage derivatization, but metal–carbon exchange is underexplored due to the challenges in selectively cleaving highly inert chemical bonds and forming stable intermediates. Here, skeletal metalation of lactams enables a carbonyl-to-nickel exchange via Ni(0) reagent-mediated selective C–N bond oxidative addition and decarbonylation, generating synthetically useful organonickel reagents for the deletion and exchange of single atoms in the lactam core.

Molecular skeletal editing relies on strong bond activation and a ‘break-to-make’ approach^1^. Existing examples include exchanging a skeletal carbon atom with X, N, O, ^13^C and B atoms, as well as its use for ring contractions and expansions. The introduction of a metal into an organic molecule using this approach has great potential for metal-mediated single-atom editing in drug-like molecules. However, metal–carbon exchange requires new disconnection approaches to directly remodel the ring structures.

Now, Bill Morandi and colleagues from ETH Zürich in Switzerland report a skeletal metalation strategy that allows metal–carbon exchange within a lactam ring by using a Ni(0) reagent under mild conditions (Fig. 1), and apply the resulting organonickel compounds for the late-stage derivatization of a bioactive azasteroid (10.1038/s41467-023-40979-3)^2^.

**Fig. 1:**
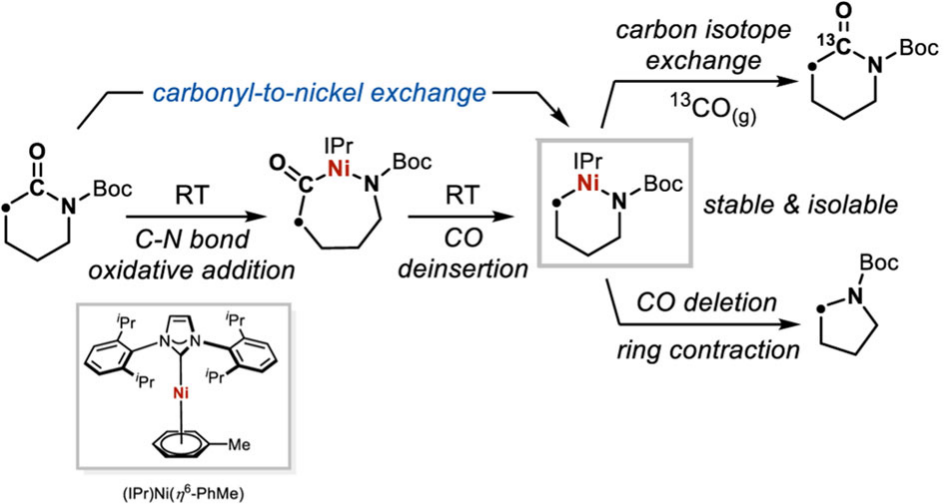
Skeletal editing of lactams by a carbonyl-to-nickel exchange strategy. An *N*-heterocyclic carbene-ligated Ni(0) complex is used to selectively activate a lactam containing an *N*-Boc amide moiety. Adapted from *Nature Communications*, **14**, 5273 (2023) 10.1038/s41467-023-40979-3.

For amide bonds specifically, skeletal activation has so far relied on transition metal-catalyzed decarbonylative coupling under harsh conditions^3^. Meanwhile, *N*-heterocyclic carbene (NHC)-ligated Ni(0) complexes have shown high activity towards inert bond activations. Here, the team find that (IPr)Ni(0)(η^6^-PhMe) can favorably bind and activate 4- to 8-membered lactam rings, allowing selective C–N bond oxidative addition and decarbonylation of *N*-Boc amides, in turn affording stable organonickel reagents for single-atom editing. Using a single-component organometallic nickel compound proves to be crucial to avoid reorganization of the metal and extra ancillary ligands in the reaction.

“Now you can take this simple Ni(0) reagent, exchange a lactam CO group for Ni, and then convert it to different bioactive scaffolds.” says Morandi. Indeed, the team developed a one-pot protocol for the skeletal metalation of azasteroid and applied it to a decarbonylative ring contraction and a carbon isotope exchange without the need to isolate intermediates.

“An ultimate goal is to turn the reaction catalytic, which would be challenging considering thermodynamics,” comments first author Hongyu Zhong. “Beyond the obvious follow-up projects, e.g. trying to access even more diverse structures from the central organonickel reagents, we hope the concept of skeletal metalation will open a new research area within the molecular editing field. Given the success of well-known metal halogen exchange reactions, we hope that our new approach to making and using organometallic reagents will have a big impact on organic synthesis,” concludes Morandi.
